# The brain basis of musicophilia: evidence from frontotemporal lobar degeneration

**DOI:** 10.3389/fpsyg.2013.00347

**Published:** 2013-06-21

**Authors:** Phillip D. Fletcher, Laura E. Downey, Pirada Witoonpanich, Jason D. Warren

**Affiliations:** Dementia Research Centre, UCL Institute of Neurology, University College LondonLondon, UK

**Keywords:** music, musicophilia, craving, frontotemporal dementia, degeneration

## Abstract

Musicophilia, or abnormal craving for music, is a poorly understood phenomenon that has been associated in particular with focal degeneration of the temporal lobes. Here we addressed the brain basis of musicophilia using voxel-based morphometry (VBM) on MR volumetric brain images in a retrospectively ascertained cohort of patients meeting clinical consensus criteria for frontotemporal lobar degeneration: of 37 cases ascertained, 12 had musicophilia, and 25 did not exhibit the phenomenon. The syndrome of semantic dementia was relatively over-represented among the musicophilic subgroup. A VBM analysis revealed significantly increased regional gray matter volume in left posterior hippocampus in the musicophilic subgroup relative to the non-musicophilic group (*p* < 0.05 corrected for regional comparisons); at a relaxed significance threshold (*p* < 0.001 uncorrected across the brain volume) musicophilia was associated with additional relative sparing of regional gray matter in other temporal lobe and prefrontal areas and atrophy of gray matter in posterior parietal and orbitofrontal areas. The present findings suggest a candidate brain substrate for musicophilia as a signature of distributed network damage that may reflect a shift of hedonic processing toward more abstract (non-social) stimuli, with some specificity for particular neurodegenerative pathologies.

## Introduction

Music is a cultural universal of human societies and the ability to appreciate music is widely prized. Music reliably evokes strong physiological as well as cognitive emotional responses (Khalfa et al., 2002; Baltes et al., 2011) and these responses have been linked to a distributed cortico-subcortical brain network that mediates biological drives and rewards and the evaluation of emotional and social signals more generally (Blood and Zatorre, 2001; Peretz and Zatorre, 2005; Omar et al., 2011). However, the neurobiological role of music and the reasons these organized abstract sounds should hold such appeal for our species remain elusive (Mithen, 2005; Warren, 2008). Recently, the musical brain has attracted considerable clinical interest, motivated by the prospect of mutually informative insights into both brain disease *per se* and the music processing brain networks that are vulnerable in particular brain diseases (Omar et al., 2012). If music processing can be targeted relatively selectively by brain damage, this lends credence to the idea that these critical brain substrates (and by implication, music itself) served an important though as yet undefined role during human evolution. The picture emerging from clinical studies, particularly in neurodegenerative dementia diseases, suggest that music (like other complex phenomena) has a modular cognitive architecture instantiated in distributed brain regions (Omar et al., 2010, 2011; Hsieh et al., 2011, 2012). In the case of music processing, the neural substrates exposed by disease are particularly extensive, including temporal and parietal areas implicated in perceptual analysis of music and musical memory, subcortical structures implicated in reward and autonomic responses and frontal lobe regions engaged in the evaluation of sensory signals and programing of an integrated behavioral response. Together, however, these diseases-associated substrates correspond closely to the coherent large-scale brain network identified in studies of music processing by the healthy brain.

Abnormally enhanced appreciation of music or “musicophilia,” reflected in increased listening to music, craving for music, and/or willingness to listen to music even at the expense of other daily life activities, may rarely signal brain disease: examples include neurodevelopmental disorders such as Williams' syndrome (Martens et al., 2010), head trauma (Sacks, 2007), stroke (Jacome, 1984), temporal lobe epilepsy on anticonvulsant therapy (Rohrer et al., 2006), and focal degenerations particularly involving the temporal lobes (Boeve and Geda, 2001; Hailstone et al., 2009). Rohrer et al. (2006) described the case of a 65 year old woman with typical temporal lobe seizures and a right temporal lobe correlate on EEG who developed selective musicophilia *de novo* after commencing anticonvulsant treatment with lamotrigine; these authors argued that musicophilia in this case was the result of altered cortico-limbic linkage in the ictal medial temporal lobe. The patient reported by Boeve and Geda (2001) became infatuated with polka music several years after onset of semantic dementia (SD) at the age of 52. Hailstone et al. (2009) described the case of a musically untrained 56 year old woman with SD who became intensely interested in music, playing, and singing along to a small repertoire of recorded pop songs; she also sang along with advertising jingles on the television. Though it might be regarded as benign in its own right, musicophilia may be highly dysfunctional when it leads to potentially deleterious music-seeking behavior, when other aspects of the patient's life suffer on account of the symptom or when it disrupts the lives of care-givers and family members (Boeve and Geda, 2001). Functional or structural alterations within the neural circuits that link cortical coding of music with evaluative and hedonic responses might plausibly give rise to musicophilia. This interpretation would be consistent both with available clinical data (Boeve and Geda, 2001; Rohrer et al., 2006; Hailstone et al., 2009; Omar et al., 2010, 2011; Hsieh et al., 2011, 2012) and with functional imaging work in the healthy brain (Blood and Zatorre, 2001; Peretz and Zatorre, 2005) implicating antero-medial and inferior frontal lobe neocortices and their subcortical connections to limbic and brainstem autonomic structures in the generation of intensely pleasurable responses to music. The phenomenon of musicophilia potentially holds unique insights into the specific, critical neural substrates that lend music its peculiar power over our species: a problem that has attracted much recent controversy (Mithen, 2005; Warren, 2008). Moreover, as a rare example of a “positive” behavioral consequence of brain damage, musicophilia may be no less informative for our understanding of disease pathophysiology. However, as a clinical phenomenon this unusual symptom has seldom been studied and the brain mechanisms that produce it remain largely undefined.

The frontotemporal lobar degenerations (FTLD) are a diverse group of dementia diseases sharing a propensity to produce selective brain atrophy predominantly involving the temporal or frontal lobes due to deposition of pathogenic proteins. Many cases have an identifiable disease-causing genetic mutation in one of three major genes (the microtubule-associated protein tau (MAPT), progranulin (GRN), and C9ORF72 genes (Rohrer and Warren, 2011). Patients typically present with one of three canonical clinical syndromes (Gorno-Tempini et al., 2011; Rascovsky et al., 2011): behavioral variant frontotemporal dementia (bvFTD), led by progressive erosion of inter-personal and executive skills; SD, led by progressive impairment of understanding of words, objects, and concepts; and progressive non-fluent aphasia, led by progressive impairment of language output with effortful misarticulated speech and agrammatism. Recent advances in molecular biology have greatly furthered our understanding of the brain bases for the development of FTLD: in particular, there is the promise of predicting specific molecular substrates from characteristic clinico-anatomical profiles, due to targeted destruction of specific large-scale brain networks by abnormal molecules (Seeley et al., 2009; Rohrer et al., 2011; Warren et al., 2012). However, to realize this promise will require an improved understanding of the sometimes complex behavioral symptoms that characterize these diseases, and in particular, how these are linked to brain network disintegration in different FTLD syndromes.

Abnormalities of emotion processing and altered social and appetitive behaviors occur in all FTLD syndromes but are particularly early and salient in bvFTD and SD (Boeve and Geda, 2001; Hailstone et al., 2009; Omar et al., 2010, 2011; Rascovsky et al., 2011). Among these behavioral abnormalities, many patients with FTLD exhibit a change in musical preferences which often takes the form of musicophilia (Boeve and Geda, 2001; Hailstone et al., 2009). In this study, we addressed the neuroanatomical basis of musicophilia in a series of patients with FTLD. Using voxel-based morphometry (VBM) of patients' MR brain images, we compared quantitatively the regional brain atrophy patterns of those who did with those who did not exhibit musicophilia. Based on available evidence from previous single cases studies (Boeve and Geda, 2001; Rohrer et al., 2006; Hailstone et al., 2009) and neuroanatomical evidence in the healthy brain (Blood and Zatorre, 2001), we hypothesized that musicophilia would be linked to increased atrophy focally involving antero-medial temporal lobe structures.

## Materials and methods

### Patients

Patients were recruited via the tertiary Cognitive Disorders Clinic at The National Hospital for Neurology and Neurosurgery. All had been diagnosed with a syndrome of FTLD (either bvFTD or SD) by a senior neurologist according to current consensus criteria (Gorno-Tempini et al., 2011; Rascovsky et al., 2011), based on detailed clinical and neuropsychological evaluation and supported by characteristic profiles of regional atrophy on structural volumetric brain MRI. Specifically, individual patients with SD showed asymmetric, focal brain atrophy predominantly involving the anterior, medial, and inferior temporal lobes; while patients with bvFTD showed predominant frontal lobe atrophy with less marked involvement of anterior temporal lobes and relative sparing of more posterior cortical areas. For the purposes of this study, patients were classified as exhibiting or not exhibiting musicophilia as defined above (musicophilic/non-musicophilic), based chiefly on retrospective review of data obtained from a research questionnaire administered to care-givers detailing patients' behavioral symptoms, including altered musical listening habits, since the onset of the clinical syndrome. Some cases were ascertained by retrospective review of clinical care-giver interviews. “Musicophilia” was defined as increased interest in music compared with the patient's premorbid behavior, as reflected in increased time spent listening to music or requests to listen to music and/or heightened music-seeking or music associated behaviors (such as dancing or singing along to music). Thirty-seven patients with a syndrome of FTLD were included in the study: 12 “musicophilic” (five with bvFTD, seven with SD) and 25 “non-musicophilic” (14 with bvFTD, 11 with SD). Seven patients with bvFTD had genetic confirmation of a pathogenic mutation causing FTLD (five cases with MAPT and three cases with C9ORF72 mutations). Patient demographic, clinical, and neuropsychological characteristics are summarized in Table 1. All patients gave written informed consent to participate in the study, which was approved by the local research ethics committee and conducted in accordance with the Declaration of Helsinki.

**Table 1:** **Summary of patient demographic, clinical, and neuropsychological characteristics**.

	**Musicophilic *n* = 12**	**Non-musicophilic *n* = 25**	***p* Value**
**DEMOGRAPHIC AND CLINICAL**			
Age (years)	62 (6.9)	64 (6.5)	n.s.
Education (years)	12.6 (2.9)	14.7 (3.1)	n.s.
Sex (F:M)	6:6	8:17	n.s.
Symptom duration (years)	6.8 (2.5)	5.0 (1.8)	0.06
Syndrome (no. of cases)			
bvFTD	5	14	
SD	7	11	
**NEUROPSYCHOLOGICAL**			
IQ			
WASI verbal	66 (26)	78.2 (23)	n.s.
WASI performance	91 (16)	86 (18)	n.s.
NART(/50)	23 (14)	24 (16)	n.s.
**EPISODIC MEMORY**			
RMT words (/50)	29 (4.5)	33 (10.4)	n.s.
RMT faces (/50)	30 (8.1)	30 (8.3)	n.s.
**SEMANTIC MEMORY**			
BPVS (/150)	69 (54.6)	103 (47.2)	n.s.
Synonyms (/50)	27 (10.1)	36.6 (14.0)	n.s.
**EXECUTIVE FUNCTION**			
Stroop inhibition (seconds)	92 (34.8)	90 (34.9)	n.s.
Reverse digit span (max)	3 (1.7)	4 (1.7)	n.s.
**SOCIAL COGNITION**			
TASIT emotion	5 (2.8)	7 (3.3)	n.s.
recognition (/14)			
TASIT social inference (/36)	17 (2.8)	23 (5.0)	0.004
**VISUOPERCEPTUAL**			
VOSP object decision	14 (3.9)	16 (3.4)	n.s.

*Mean (standard deviation) data are shown where appropriate. BPVS, British picture vocabulary scale; NART, national adult reading test; n.s., non-significant; RMT, recognition memory test; TASIT, the awareness of social inference test; VOSP; visual object and space perception battery; WASI, Wechsler abbreviated scale of intelligence*.

### Brain image acquisition and pre-processing

At the time of behavioral assessment, all patients underwent brain MRI on a 3T GE Signa scanner (General Electric, Milwaukee, WI, USA) using a 12 gage head coil. T1 weighted images were obtained with a 24 cm field of view and 256 × 256 matrix to provide 124 contiguous 1.5 mm thick slices in the coronal plane 9 echo time (TE) = 5 ms, repetition time (TR) = 512 ms, inversion time (TI = 5650 ms). Pre-processing of patients' MR images was performed using the DARTEL toolbox of SPM8^1^ running under MATLAB 7.0^2^. Normalization, segmentation, modulation, and smoothing of gray and white matter images were performed using default parameter settings. In order to adjust for individual differences in global gray matter volumes during subsequent analysis, total intracranial volume (TIV) was calculated for each patient by summing gray matter, white matter, and cerebrospinal fluid volumes following segmentation of all three tissue classes.

### VBM analysis

Voxel-based morphometry analysis of brain images was based on a linear regression design in SPM8, modeling voxel intensity as a function of the presence or absence of musicophilia across the patient group. Patient age, gender, TIV, and clinical syndromic group were included a covariates of no interest. A customized explicit brain mask was applied based on specific consensus voxel threshold intensity criterion including all voxels with intensity >0.1 in >70% of subjects. Statistical parameter maps (SPMs) of regional gray matter volume contrasting the musicophilic and non-musicophilic subgroups were examined at a threshold of *p* < 0.05 after family wise error (FWE) corrections for multiple comparisons over the whole brain and after small volume correction based on our priori anatomical hypothesis. Generous anatomical small volumes were created separately for the left and right anterior temporal lobes by manually tracing from the template brain image using MRICron^3^ each small volume comprised the antero-medial temporal lobe anterior to Heschl's gyrus.

## Results

### Clinical and behavioral characteristics

The musicophilic and non-musicophilic patient subgroups did not differ in mean age, gender, or years of education (Table 1); average disease duration was non-significantly longer (*p* = 0.06) in the musicophilic subgroup. On neuropsychological evaluation, the musicophilic subgroup was significantly more impaired (*p* < 0.004) than the non-musicophilic subgroup on a test of social cognition (the Awareness of Social Inference Test social inference subtest); the subgroups performed similarly on tests of general executive function, memory, and visuoperceptual skills (Table 1). Musicophilia developed more frequently in the SD syndromic group (39% of cases) than the bvFTD syndromic group (26% of cases). The proportion of patients with musicophilia was similar among cases with particular genetic mutations versus sporadic cases (one patient with a MAPT mutation and one with a C9ORF72 mutation in the musicophilic subgroup; other genetic cases in the non-musicophilic group).

Details of changes in patients' music listening behavior based on care-giver comments are summarized in Table A1 in Appendix. Qualitatively, most patients in the musicophilic subgroup spent more time listening to music. In several cases, musicophilia was accompanied by a change in musical preferences (for example, from classical or jazz to pop or church music). Musicophilia was often accompanied by complex behaviors, such as watching music videos for much of the day or singing and dancing along to the music. However, the salience of musicophilia (for example, the amount of time spent listening to music each day or the intensity and intrusiveness of music-seeking behaviors) varied widely among individual patients who exhibited the phenomenon. Most patients in the non-musicophilic subgroup had no change in their premorbid music listening behavior, however there were several who had lost interest in music or developed an active aversion to music following the onset of cognitive decline. None of the patients with musicophilia was a professional musician; however, detailed data on patients' premorbid musical training or experience were not available.

### Neuroanatomical associations

**Figure 1:**
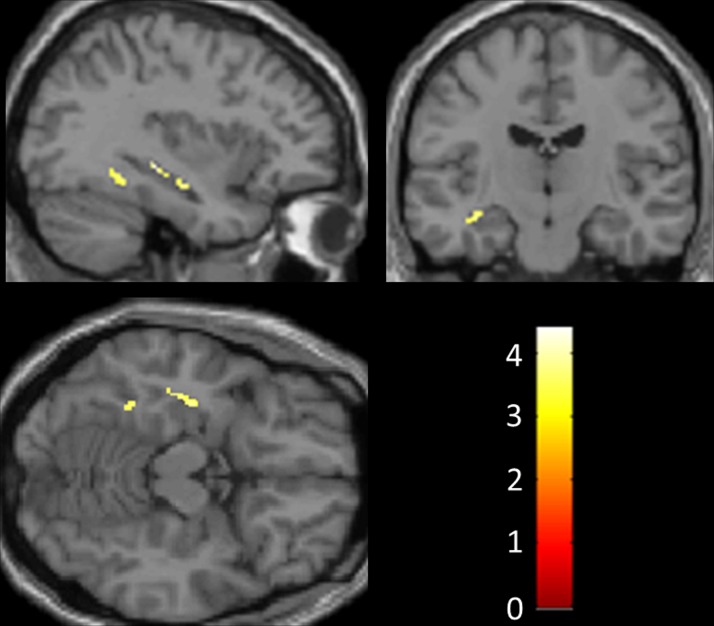
**Statistical parametric maps of regions of significant gray matter preservation in the musicophilic relative to the non-musicophilic patient subgroup (shown at an uncorrected threshold *p* < 0.001; atrophy of left hippocampus significant at *p* < 0.05 after small volume correction for multiple voxel-wise comparisons)**. All gray matter correlates with cluster size >20 voxels are shown. SPMs are displayed on sagittal (above left), coronal (above right), and axial (below left) sections through the anterior temporal lobes from a canonical T1 weighted brain template image in Montreal Neurological Institute standard stereotactic space. The sagittal section is through the left cerebral hemisphere; the coronal section shows the left hemisphere on the left. *Z* scores are coded on the color bar (below right).

No regional gray matter differences were found between the two patient subgroups (*p* < 0.05) after correction for multiple voxel-wise comparisons over the whole brain volume. However, the musicophilic subgroup showed significantly increased regional gray matter volume relative to the non-musicophilic group in left posterior hippocampus (*p* < 0.05) after small volume correction over the anterior temporal lobe volume of interest (Figure 1; Table 2). At a less stringent uncorrected threshold *p* < 0.001 over the whole brain volume, additional regional gray matter associations of musicophilia (relative to the non-musicophilic patient subgroup) were identified in left parahippocampal gyrus, temporo-parietal junction and anterior cingulate, and bilateral dorsolateral prefrontal cortices (Table 2). Conversely (also at an uncorrected threshold *p* < 0.001 over the whole brain volume), the musicophilic subgroup showed significantly reduced regional gray matter volume than the non-musicophilic group bi-hemispherically in posterior parietal cortex, medial orbitofrontal cortex, and frontal pole (Table 2).

**Table 2:** **Summary of voxel-based morphometry findings**.

**Group gray matter correlate**	**Brain region**	**Side**	**Local max (mm)**			***Z*-score**	**Cluster size (voxels)**
			**x**	**y**	**z**		
Musicophilic > non-musicophilic	Posterior hippocampus^*^	L	−36	−28	−8	3.86	141
	Temporo-parietal junction	L	−44	−49	36	3.87	36
	Dorsolateral prefrontal cortex	R	21	8	48	3.59	31
		L	−21	12	45	3.49	37
	Anterior cingulate	L	−8	24	21	3.50	27
	Parahippocampal gyrus	L	−33	−45	−15	3.38	56
Musicophilic < non-musicophilic	Posterior parietal cortex	R	24	−82	32	4.66	438
		L	−18	−76	45	3.65	54
	Frontal pole	L	−6	69	12	4.10	217
		R	24	66	−2	3.74	116
	Medial orbitofrontal cortex	L	−4	66	−17	3.93	151
		R	22	64	−14	3.50	59
	Dorsolateral prefrontal cortex	R	36	56	24	3.69	67

*Cerebral gray matter regions significantly preserved (above) or atrophic (below) in the musicophilic subgroup relative to the non-musicophilic subgroup are listed with peak coordinates in Montreal Neurological Institute standard stereotactic space and associated cluster sizes. All gray matter correlates with cluster size >20 voxels are shown*.

* *Significant at threshold p < 0.05 corrected for multiple comparisons within the prespecified anatomical small volume of interest (see text); other results significant at p < 0001 uncorrected over the whole brain volume*.

## Discussion

Here we describe a candidate brain substrate for the symptom of musicophilia developing in the context of degenerative brain disease. Comparing subgroups of patients with FTLD that were well matched for other clinical and neuropsychological characteristics, development of musicophilia was specifically associated with relative preservation of gray matter in posterior hippocampus and (less robustly) a distributed network of additional areas including parahippocampal, temporo-parietal, anterior cingulate, and prefrontal cortices; and with atrophy of gray matter in posterior parietal and orbitofrontal cortices. Consistent with these neuroanatomical findings and with the previous clinical literature (Boeve and Geda, 2001; Hailstone et al., 2009), musicophilia was more commonly associated with the syndrome of SD (associated with focal antero-medial temporal lobe and inferior frontal lobe atrophy) than bvFTD; however, it is unlikely the neuroanatomical associations of musicophilia we observed were driven simply by these syndromic groupings, since the associations were detected after covarying for syndromic membership. The present anatomical findings corroborate previous reports that focal alterations of hippocampal function can give rise to musicophilia (Rohrer et al., 2006), and further affirm the role of the anterior temporal lobes in processing dimensions of music in FTLD (Hsieh et al., 2011, 2012; Omar et al., 2011, 2012).

Although the anatomical correspondence was not precise, it is of interest that gray matter areas relatively preserved in our musicophilic group overlapped with those previously associated with the “default mode network” that has been proposed to mediate internally directed thought as well as the pathogenesis of another neurodegenerative illness, Alzheimer's disease (Pievani et al., 2011). Areas of greater gray matter atrophy in the musicophilic group also overlapped the “salience” network previously implicated in social cognition and more specifically understanding of others' mental states, including mental states as represented in music (Seeley et al., 2009; Downey et al., 2012). In addition, the network of areas we have demonstrated includes a number of brain regions previously implicated in mediating musical memory and emotional responses to music in the healthy brain (Platel et al., 2003; Koelsch et al., 2006; Watanabe et al., 2008; Herholz et al., 2012), while altered connectivity within this network may provide a mechanism for impaired acquisition of musical skills in congenital amusia (Hyde et al., 2006, 2011) and for acquisition of skills during musical training (Groussard et al., 2010). Such previous observations suggest that this brain network can modulate the experience of music in response to various developmental and acquired factors: the present neuroanatomical data further suggest that the activity of the network is susceptible to neurodegenerative brain disease. The present behavioral data indicate that musicophilia may be associated with relatively greater impairment of inter-personal social inference (see Table 1): considered together with the neuroanatomical findings, we propose that abnormal craving for music in this patient population is a marker for concomitantly less efficient interpretation of social signals; and more speculatively, for a shift toward the more abstract hedonic valuation that music represents. Initially, this might seem somewhat surprising in view of the widely recognized social role of music and previous arguments advanced by our group and others in support of a role for music in modeling surrogate social interactions (Mithen, 2005; Warren, 2008; Downey et al., 2012). However, in addition to any social attributes it acquires, music has the properties of a self-contained, abstract, non-referential meaning system with “rules” that must be learned by the members of a particular musical culture (Omar et al., 2010). The rhythmic and melodic attributes of music establish an internal sense of expectation and resolution which may carry its own cognitive reward (Meyer, 1956; Huron, 2006). The present data do not resolve the mechanism whereby music can acquire abnormally high emotional value for cognitively impaired patients. We propose, however, that this may reflect a skewed balance between relatively intact processing of musical signals and a relatively intact capacity to link these signals with autonomic and other internal states, versus degraded hedonic processing of social and other environmental signals. Music might therefore be somewhat analogous to other categories of abstract stimulus (for example, number puzzles) in which patients with FTLD may also show obsessional interest. A further analogy might be drawn with the often preserved musical capacities of individuals with autism despite markedly impaired social signal processing (Molnar-Szakacs and Heaton, 2012), with a number of similarities to the behavioral syndromes of FTLD.

We do not argue that musicophilia is a universal marker of FTLD pathology: across our FTLD cohort, individual patients showed wide variation both in the extent and indeed the direction of their hedonic shift in response to music. This situation is somewhat reminiscent of the individual variation in musicality described among individuals with Williams' syndrome (Martens et al., 2010), or the behavioral heterogeneity of the dopamine dysregulation syndrome in Parkinson's disease (Merims and Giladi, 2008). The latter has been linked to dysfunction of distributed neural circuits including basal forebrain, limbic, and prefrontal cortical areas: interestingly, while a wide variety of addictive behaviors have been described, musicophilia appears to be uncommon (or perhaps under-reported as relatively benign). The sources of individual susceptibility to addictive behaviors in these conditions largely remain to be defined; however, we believe it is unlikely that musicophilia simply reflects the relative premorbid importance of music in patients' lives, as several of our cases with prominent musicophilia had no formal musical training. We hypothesize that the phenomenology of the behavior may have some specificity for the underlying neural substrate for the disease group as a whole; and in particular, that the development of musicophilia in FTLD is a novel behavioral signature of the salience and semantic networks previously implicated in the pathogenesis of FTLD (Seeley et al., 2009). However, it is important to recognize that musicophilia is part of a much wider repertoire of abnormal behaviors that emerge in FTLD, including other behaviors with obsessional or ritualistic features (Rascovsky et al., 2011). How musicophilia relates to this spectrum remains to be defined.

This study has several limitations that suggest direction for future work. Patient numbers here were relatively small, and behavioral testing was limited due to the retrospective nature of the case ascertainment: further work in larger cohorts should address the phenomenology and brain substrate of musicophilia prospectively and quantitatively, incorporating physiological measures of arousal and attempting to quantify the expression of music craving. In order to fully understand this phenomenon, it will be necessary to determine how musicophilia relates to general musical competence and esthetic evaluation; our purely clinical impression is that musicophilia in the present and previous cases (Boeve and Geda, 2001; Hailstone et al., 2009) was often accompanied by loss of prior musical discrimination, and these aspects might be integrally associated. It will be important to assess musicophilia in relation to abnormal extra-musical behaviors associated with FTLD. The true frequency of musicophilia remains unknown: future work should investigate other disease groups as well as FTLD, ultimately with histopathological correlation. Inferences that can be drawn from VBM studies are essentially associational: the gray matter changes identified here may not be necessary or sufficient to produce musicophilia. The specific brain mechanism of musicophilia might however be defined in future using functional MRI paradigms that compare brain responses to music versus other complex (and potentially arousing) auditory stimuli. We hope that the present findings will motivate further systematic behavioral and neuroanatomical investigation of this intriguing phenomenon.

### Conflict of interest statement

The authors declare that the research was conducted in the absence of any commercial or financial relationships that could be construed as a potential conflict of interest.

## Footnotes

^1^www.fil.ion.ucl.ac.uk/spm

^2^www.matlab.org

^3^www.mccausandcentre.sc.edu/micro/micron

## Appendix

**Table A1:** **Summary of changes in music listening in patient subgroups**.

**Group**	**Data source**	**Care-giver comments**
Non-musicophilic *N* = 25	Questionnaire *n* = 17	No change
	Questionnaire *n* = 6	Can only tolerate music for short periods now
		Used to like jazz and classical music, now cannot identify favorite singers
		Listens to music less now
		No interest in listening to music
		Plays less music at home
		Used to love to play CDs but never puts them on now
	Care-giver interview *n* = 2	No change
Musicophilic *N* = 12	Questionnaire *n* = 9	Loves all types of music more than he used to
		Now loves singing and dancing along to songs she knows
		Listens to music a lot more now
		Listens to music a lot more now
		Less interest in classical music but listens to music more, now favors 1970s rock from his youth
		Listens endlessly to music on his i-pod but is sensitive to other noise such as children playing
		Listens to music more than he used to and wears his i-pod all the time
		Music makes her dance, it makes her want to dance and she likes listening to it more
		Plays music louder, likes to play more music, sings along, listens to ABBA for hours now
	Care-giver interview *n* = 3	Likes music more, plays the same song repeatedly and will dance to music at home by herself
		Developed a habit of singing along to music on CDs or television
		Rekindled interest in music, started attending church and singing hymns
